# Supplementary material for: Development and validation of context-specific components of obstetric violence: Experiences from the central zone of Tanzania
Source: PLoS One. 2025 Jun 18;20(6):e0326362. doi: 10.1371/journal.pone.0326362 (PMC12176237; doi:10.1371/journal.pone.0326362)
Supplement: Attachment — Submitted filename: Review 2.pdf [file pone.0326362.s003.pdf]

Note that I maintain my previous responses based on the publication criteria and responses.

Development and validation of context-specific components of obstetric violence: Experiences from Central Zone Tanzania

This peer review has been finalized based on the following sub-topics as per the publication criteria.

1. **Originality of the study and the results**

The study clearly presents the topic which in my view aligns well with a clear goal, methodology, results, limitations and conclusions. I also noted that the study has a good introduction. However, my suggestion is that the authors can consider enriching this introduction with global, regional and national statistics and rates to show the burden of obstetric violence and hence contributing to a clear case for action.

2. **Results reported have not been published elsewhere**

I have done a search for the topic but I have not found any record of the results having been published anywhere. This is a clear reflection of originality of the results.

3. **Experiments, statistics, and other analyses are performed to a high technical standard and are described in sufficient detail.**

The goal of developing contextual components of obstetric violence in Central Tanzania was achieved by coming up with clear conceptualization as indicated by Figure 1 which demonstrates prototype 1 and 2. I take note that the iterative-mixed methods that was utilized in the development and validation of the contextual components of obstetric violence is well described in sufficient details. Prototype 1 which involved the review of related literature to identify the typology of obstetric violence from literature is intelligently done. The identified components of obstetric violence were then validated by experts through a plenary discussion based on their applicability to the Tanzanian context. Experts' additional components of obstetric violence-based on the WHO evidence on mistreatment were included. Prototype 1 components that were validated by experts were then translated from English to Kiswahili by linguistics experts, and validation from the University of Dodoma before the face validity testing with post-natal mothers and healthcare providers. Items also underwent adjustments – a process that involved elimination of some items, merging others with similar concepts and adding others which were deemed relevant to the Tanzanian context.

Descriptive statistics that were used to summarize the experts, and demographic characteristics of postnatal mothers who participated in the study were appropriate and clearly presented in tabular form. This approach was used for data cleaning to assess data completeness and accuracy prior to qualitative analysis. The qualitative analysis of the data that was collected from experts and postnatal mothers was clearly coded, then the related codes were organized into subcategories and then sub-categories were merged to form categories which constituted items.

It is also clear that the authors used descriptive analysis to arrive at frequencies and percentage of the responses of the Likert scales based on the participants' responses. This approach was used for identification of most common recommendation in each component. High frequency recommendations were utilized in items adjustments. Each of the items were subjected to Item-level Content Validity Index computation which was arrived by dividing the total number of experts who agreed with items in terms of adequacy and its contextual relevance by the total number of experts involved. Authors are clear that they used this method as one of the criteria for item amendment. Items utilized an I-CVI value of 0.78 or higher as a consideration for content validity. It is clear that researchers arrived at a content validity values of 0.791 and 0.958.

Item face validity index (I-FVI) was computed by diving the total number of postnatal mothers and healthcare workers who agreed with the comprehensiveness and clarity of items by the total number of postnatal mothers and healthcare workers who participated in the face validation process. An I-FVI value of 0.75 or more was considered a good score and provided a good into how postnatal mothers and healthcare providers interpreted and responded to the components. For this study the I-FVI ranged between 0.77 and 0.925.

Based on the above analysis seven contextual components of OV were arrived namely: physical violence (I-CVI= 0.937), lack of supportive care and treatment (I-CVI=0.96), subjugation care (I-CVI=0.833), unfavorable care environment(I-CVI = 0.958), sexual violence(I-CVI=0.833), verbal violence (I-CVI=0.833), emotional and psychological violence (I-CVI=0.96).

Conclusion: I agree that experiments, statistics, and other analyses are performed to a high technical standard and are described in sufficient detail.

4. Conclusions are presented in an appropriate fashion and are supported by data.

The study concludes that, the validated components of obstetric violence components consisted of seven main categories that were related to health facilities and the community. The conclusion also indicated that the components achieved acceptable validity testing, hence arriving at the conclusion that the seven components were the valid contextual components that can be utilized in development of obstetric violence measurement tools in Tanzania. Authors also recommended further strengthening of the seven components by further studies.

Conclusion: I agree that the conclusion is presented in appropriate version, and are supported by data.

5. The article is presented in an intelligible fashion and is written in standard English.

I agree with this statement. The article is written and clear standard English. However, there are some minor grammatical and punctuation issues which I suggest to the authors to consider correcting as detailed hereunder.

Introduction

Globally, estimates of obstetric violence ranges from 15% to 99% (3) with prevalence in Sub-Saharan Africa reported to be between44% and 52%, In Tanzania, obstetric violence remains a significant concern (4,5). – Page 3.

Issue

Space needed between the word 'between and 44%.

Despite ongoing research in examining the definition, root causes and methodologies for assessing obstetric violence occurring during pregnancy, childbirth and after childbirth, there is no universal consensus on how to define and measure obstetric violence (8).Cultural and… Page 3.

Issue

Space between the full stop at citation 8 and Cultural needed.

Tanzania's guidelines have outlined the typology of disrespect and abuse in maternity care such as non-dignified care, abandonment, physical abuse, non-confidential care and non- consented care (10), yet they large draw from external context that may not fully reflect local realities (8,9,11). Page 4

Issue

The word "large" should be replaced with the word "largely"

Comprehensive Emergency Obstetric and New-born Care (CEmONC) (13,14) Page 4.

Issue

Full stop needed at the end of citation 13 and 14.

Maternal health care provision remains a major challenge in developing countries such as Tanzania resulting in ongoing high maternal mortality (15) .One major risk factor… page 4

Issue

Full stop needed at the end of citation 15 needed and spacing before the word, "One"

Factors linked to home births in Tanzania encompass the hesitancy to seek care at a birthing facility due to individual and community experiences of obstetric violence at the facility level as a result , some women avoid facility birth and then experience preventable birth complications hence contributing to prevailing high maternal and neonatal mortality (17) .

Issue
Space between the word result and some to be deleted.
space between citation 17 and full stop to be removed. – check the rest of the citations for similar citation issues for the rest of the text.

This study was conducted in two regions in the central zone of Tanzania from July 2023 to June 2024 .

Issue
space between citation between 2024 and full stop to be removed.

It is the regions with -----

Issue

The word “regions” to be replaced with the word “region”

DISCUSSION

Obstetric violence is globally recognized as a serious issue threatening maternal health and well-being, but there is no consensus on how best should be defined due to cultural and geographical differences (18).

Issue
The word “it” to be inserted between best and should…

Furthermore, feedback from maternal health experts, health care providers and postnatal mothers during the validation process were utilized and incorporated whereby some items were modified .

Issue

The space between modified and full stop to be removed These results align with a study conducted in the West Bank Palestine, where obstetric violence measurement tools were developed and validated, achieving a content validity index greater than 0.83 (41) indicating that all the components were valid and cultural acceptable .

Issue
The space between the word acceptable and full stop to be removed.

significant spiritual element of the childbirth experience (45) .

Issue
Space between citation 45 and the full stop to be removed.

There was particular concern about managing mentally distressed women during labor , considering staffing constraints.

Issue

Letter ‘a’ to be inserted between the words “was” and “particular”.

restraint was unnecessary in any situation and they perceived it as a display of healthcare providers lacking empathy (50) .

Issue

Spacing issues as highlighted in earlier comments.

… involves non-consensual sexual acts against pregnant women whether in community or healthcare settings (51–53)

Issue

Full stop needed after citation 51-53.

6. **The research meets all applicable standards for the ethics of experimentation and research integrity.**

I agree with this statement. Researchers proved that all applicable standards for ethics and of experiments and research integrity were met by the researchers.

7. **The article adheres to appropriate reporting guidelines and community standards for data availability.**

I agree that the article meets appropriate reporting guidelines and community standards for data availability.
