# Supplementary material for: Development and validation of context-specific components of obstetric violence: Experiences from the central zone of Tanzania
Source: PLoS One. 2025 Jun 18;20(6):e0326362. doi: 10.1371/journal.pone.0326362 (PMC12176237; doi:10.1371/journal.pone.0326362)
Supplement: Attachment — Submitted filename: PLOS ONE Review.docx [file pone.0326362.s001.docx]

**PLOS ONE Review: Development and validation of context-specific components of obstetric violence: Experiences from Central Zone Tanzania**

This important study investigates components of obstetric violence (OV) in the Central Zone of Tanzania using a mixed-methods approach with a variety of participants, including postnatal mothers, healthcare providers and community members, ultimately identifying seven categories of OV, with acceptable levels of item-level content validity and item-face validity. The manuscript suffers from some distracting grammatical errors and could benefit from further conceptualization of the practical implications of the tool, but with some fine-tuning, this work could contribute importantly to the growing literature on OV.

A few grammatical issues to be addressed throughout the paper: capitalizations (many words are unnecessarily capitalized), comma placement/usage (there are commas throughout the manuscript with multiple spaces before and after them and places where commas are missing where needed, particularly for embedded phrases), lack of periods at the end of sentences, subject/verb agreement. Additional edits suggested below.

Abstract:

Methods:

- The sentence in methods beginning with “The design involved...” is confusing, please clarify. How does the literature review fit into this picture? Consider removing this sentence and listing out the steps of the study sequentially.
- Edit: “they included ‘a’ gynecologist or gynecologist’s’; an obstetrician or obstetrician’s’.
- The term “end users” is confusing here. Please clarify. How will this be “used”?

Results:

- Please remove capitalization of “Unfavorable.”

Conclusion:

- This sentence is confusing and the verb tenses don’t line up. It may also be a fragment. Please re-arrange with concise and strong sentence.

Introduction:

- P. 3, first sentence: consider editing to: “while pregnant, during labor, and after childbirth.”
- P. 3, second paragraph, last phrase: please explain what you mean by, “should be analysed from a different context.” (or remove that phrase)
- Please clarify that these are the UN’s SDGs, then relate these goals back to obstetric violence.
- P. 3, 3^rd^ paragraph: given that obstetric violence is a relatively new concept, I would recommend removing the phrase, “While the forms and underlying causes of obstetric violence are now extensively recorded.” Also consider combining this paragraph with the next one, which could be shortened to just its final sentence.
- P. 4, 2^nd^ paragraph, first sentence starting with “In Tanzania,” this sentence is difficult to follow, consider breaking it up. Same for the sentence beginning with “Without acknowledging…”—it is unclear how this could lead to over-reporting.

Material and Methods:

- P. 4-5: Please add period after end of first paragraph.
- P. 5: First full paragraph: Please either add commas around “Tanzania being one of them,” or rearrange the first sentence. Also, consider providing additional support to the last sentence of this paragraph. This is a very important point, consider adding at least one more sentence that lays out the connection between obstetric violence and maternal mortality. Also please add a period at the end of this paragraph.
- Please explain the process for going from focus group and interviews into the creation of the prototype. What kind of analysis was done? By whom?

Phase III

Participants:

- P. 10- Please remove from first sentence: “if they are contextually valid.”
- P. 10- Consider adding the numbers of participants for each category of expert.

The workshop:

- P. 10- Please clarify the criteria the experts are using to rate the components. Were they rating whether or not these components should be included in the model? Or if these factors are important to their patients? Or which items they’ve had experience with? Please list the specific prompt.
- P. 10- Please clarify how the experts provided feedback on the applicability of the component to the Tanzania context. Was this open-ended feedback?

Phase V: Face validity testing

- P. 11- Same comment as above—please include the prompt the women were responding to on the Likert scale.

Analysis

- P. 13- Please clarify your criteria for considering recommendations with a “high prevalence.” How did you determine the cut-off for high-medium-low?
- P. 13- Please justify your use of 0.78 as your cut-off for the I-CVI and a cut-off of 0.75 for I-FVI.

Results:

Maternal Health Experts:

- P. 14- The age group category in Table 2 is confusing as presented. Is there something you are trying to demonstrate with this variable? If this is meant to show that some of these groups are younger than others, there may be a more effective way to show that. If it is just to provide context, average age may be preferrable.

Physical violence:

- P. 21- Please provide further explanation for “Kicking was reported as impractical.” Were these items assessed for practicality?

Lack of supportive care and treatment:

- P. 21- Please spell out what the acronym ANC stands for the first time it appears in the text.

Sexual violence:

- Please provide further support for removing the category of rape. Provide additional support for this sentence: “The discussion led to the consensus that rape is not just an act of violence but a criminal offense, even though there have been reports of such actions by healthcare providers.” How did rape’s status as a crime fit into the discussion? What were the primary reasons laid out by the group for this item’s removal?
- P. 23- Please clarify the sentence that begins with the phrase, “Some postnatal women…” Does this mean postnatal women suggested adding a new category: “to be forced to have sex with their partner during pregnant even when they are not feeling well”? Please explain, “claiming they are warm.”

Psychological/emotional violence:

- P. 23- Please add the word “differently” to the following item: “treating a woman (differently) based on her age, parity and marital status…”
- P. 23- Please clarify this phrase, “experts and postnatal mothers added teenagers and high parity women as they are so vulnerable to violence during care provision at the health facilities in our settings.” Does this mean additional items based specifically on age and parity were added to the prototype?

Discussion

- P. 26- Please clarify the sentence beginning with, “Experts also recommended the inclusion Health…” It is difficult to follow.

Strength and limitation

- P. 27- Add a strength that highlights the practical utility of this OV prototype—what can it do, how can it advance the field?
- P. 27- Please clarify this limitation. Is there any reason to assume that male partners and/or religious leaders will have more to say on OV than your current participants?
- Consider including a limitation that addresses the difficulty of including all perspectives in one prototype for OV. How did you resolve scenarios of disagreement among your participants? How did you ensure that all of the voices in the focus groups were heard? Is it possible that relations of power played out in the focus groups, such that some voices were louder and therefore had outsized impacts on the resulting model?

Conclusion

- P. 27- Please lay out some of the more specific practical implications of your study—how can this be used to help pregnant people? To help healthcare providers? To address maternal mortality?
- Please lay out the next steps for researching this topic—what are the important questions that remain about OV in this context? What are the important areas for future research?
