# Supplementary material for: Development and validation of context-specific components of obstetric violence: Experiences from the central zone of Tanzania
Source: PLoS One. 2025 Jun 18;20(6):e0326362. doi: 10.1371/journal.pone.0326362 (PMC12176237; doi:10.1371/journal.pone.0326362)
Supplement: Attachment — Submitted filename: RESPONSE TO REVIWERS COMMENTS OV Validation.docx [file pone.0326362.s004.docx]

POINT-BY-POINT RESPONSE TO EDITOR AND REVIWERS COMMENTS

Dear editor, we would like to appreciate the comments given by you and reviewers on the manuscript titled ‘Development **and validation of context-specific components of obstetric violence: Experiences from Central Zone Tanzania’.**  As indicated below, are the comments together with the amendment made

**Reviewers’ responses.**

**Reviewer #1: Thank you for reviewing our manuscript and for the constructive comments**

| SN | Comments | Response |
| --- | --- | --- |
| 1 | This important study investigates components of obstetric violence (OV) in the Central Zone of Tanzania using a mixed-methods approach with a variety of participants, including postnatal mothers, healthcare providers and community members, ultimately identifying seven categories of OV, with acceptable levels of item-level content validity and item-face validity. | We appreciate this recognition |
| 2 | The manuscript suffers from some distracting grammatical errors and could benefit from further conceptualization of the practical implications of the tool, but with some fine-tuning ; this work could contribute importantly to the growing literature on OV.  A few grammatical issues to be addressed throughout the paper: capitalizations (many words are unnecessarily capitalized), comma placement/usage (there are commas throughout the manuscript with multiple spaces before and after them and places where commas are missing where needed, particularly for embedded phrases | The whole document has been edited for grammar and spelling errors |
| 3 | **Abstract** |  |
|  | **Methods:**   - The sentence in methods beginning with “The design involved...” is confusing, please clarify. How does the literature review fit into this picture? Consider removing this sentence and listing out the steps of the study sequentially. - Edit: “they included ‘a’ gynecologist or gynecologist’s’; an obstetrician or obstetrician’s’. - The term “end users” is confusing here. Please clarify. How will this be “used | - The sentence has been revised and the steps of the study have been sequentially written and explained in detail in the methods section of the manuscript page 5-13 - The term end user ,has also been edited to targeted users referring to pregnant/postnatal women and health care providers |
|  | **Results:**  Please remove capitalization of “Unfavorable | The capitalization have been removed |
|  | **Conclusion:**   - This sentence is confusing and the verb tenses don’t line up. It may also be a fragment. Please re-arrange with concise and strong sentence. | The whole paragraph has been re-written .Page 2-3 |
| **4** | **Introduction** |  |
|  | Introduction:   - P. 3, first sentence: consider editing to: “while pregnant, during labor, and after childbirth.” - P. 3, second paragraph, last phrase: please explain what you mean by, “should be analysed from a different context.” (or remove that phrase) - Please clarify that these are the UN’s SDGs, then relate these goals back to obstetric violence. - P. 3, 3^rd^ paragraph: given that obstetric violence is a relatively new concept, I would recommend removing the phrase, “While the forms and underlying causes of obstetric violence are now extensively recorded.” Also consider combining this paragraph with the next one, which could be shortened to just its final sentence. - P. 4 , 2^nd^ paragraph, first sentence starting with “In Tanzania,” this sentence is difficult to follow, consider breaking it up. Same for the sentence beginning with “Without acknowledging…”—it is unclear how this could lead to over-reporting. | - The sentence has been corrected as suggested ,page 3 - The whole paragraph has been rephrased and some of the phrase have been removed. Page 3 of the clean manuscript - More clarifications about the UN’s SDGs (goal 3 and 5) and how they relate to obstetric violence, has been added in page 3-4 of the clean manuscript - The whole paragraph three, was rephrased and combined with the one talking about how culture and geographical factors influence obstetric violence - These two paragraph have been rephrased and some of the phrased have been removed. Page 4-5 |
| **5** | **Material and Methods** |  |
|  | - P. 4-5: Please add period after end of first paragraph. - P. 5: First full paragraph: Please either add commas around “Tanzania being one of them,” or rearrange the first sentence. - Also, consider providing additional support to the last sentence of this paragraph. This is a very important point, consider adding at least one more sentence that lays out the connection between obstetric violence and maternal mortality. Also please add a period at the end of this paragraph. - Please explain the process for going from focus group and interviews into the creation of the prototype. What kind of analysis was done? By whom? | - The cited maternal mortality rate year has also been added page 5-6 - Additional information have been provided, showing the connection between obstetric violence and maternal mortality in page 5-6 and their periods - More description about the process for going from focus group and interviews into the creation of the prototype and the kind of analysis that was done in the qualitative phase has been added in page 7-8 |
|  | Phase III  Participants:   - P. 10- Please remove from first sentence: “if they are contextually valid.” - P. 10- Consider adding the numbers of participants for each category of expert. | - The first sentence have been edited as suggested - The number of participants for each category have been added in page 11 |
|  | The workshop:   - P. 10- Please clarify the criteria the experts are using to rate the components. Were they rating whether or not these components should be included in the model? Or if these factors are important to their patients? Or which items they’ve had experience with? Please list the specific prompt.   P. 10- Please clarify how the experts provided feedback on the applicability of the component to the Tanzania context. Was this open-ended feedback? | - Some of the criteria used by the experts to rate the components of OV included: relevance, clarity and understandability, cultural sensitivity, applicability in a Tanzanian context, and fairness ensuring no bias toward any particular group. - Each of the experts independently reviewed the components and provided written feedback. More information about this have been added to the manuscript in page 11-12 |
|  | Phase V: Face validity testing   - P. 11- Same comment as above—please include the prompt the women were responding to on the Likert scale. | - Some of the prompts that postpartum women were responding to on the Likert scales included; the clarity of wording, simplicity of the language used and understand ability, cultural appropriateness and perceived value of each component .More description has been also added to the main manuscript in page 13 |
|  | Analysis   - P. 13- Please clarify your criteria for considering recommendations with a “high prevalence.” How did you determine the cut-off for high-medium-low? - P. 13- Please justify your use of 0.78 as your cut-off for the I-CVI and a cut-off of 0.75 for I-FVI. | - The criteria for considering a recommendation with high prevalence included the frequency of responses from experts, shared experiences among the experts, the significance of the component within the Tanzanian context and current evidence from other studies and reports referenced during the workshop. More description has been also added in page 14 - The cut-off points were determined based on expert judgment and consensus. A component was considered "high prevalence" if accepted by 75% or more of the experts, "medium prevalence" if accepted by 50%-74%, and "low prevalence" if accepted by less than 50% of the participating experts. This scoring system was also used by other studies. More description has been provided to the main manuscript in page 17 - Items with an I-CVI value of 0.78 or higher were considered to possess strong content validity that indicates that at least 78% of experts agreed that an item was relevant. Page 14- 15 - An I-FVI value of 0.75 or more was considered a good score signifies that at least 75% or more of respondents find the components clear and understandable. Page 15 |
| **6** | **Results** |  |
|  | Maternal Health Experts:   - P. 14- The age group category in Table 2 is confusing as presented. Is there something you are trying to demonstrate with this variable? If this is meant to show that some of these groups are younger than others, there may be a more effective way to show that. If it is just to provide context, average age may be preferable. | - Table 2 has been amended and the mean age has been re-calculated. Page 16 |
|  | Physical violence:   - P. 21- Please provide further explanation for “Kicking was reported as impractical.” Were these items assessed for practicality? | - Thank you for your thoughtful comment. After conducting a further review and additional consultations regarding “kicking to be impractical”, all authors have agreed that the item should be retained as part of the item on physical violence that may occur at both the community level and within facilities, as there have been reported cases of such incidents. The item has been added in table 4 page 19 - The word impractical was mistakenly used |
|  | Lack of supportive care and treatment:   - P. 21- Please spell out what the acronym ANC stands for the first time it appears in the text. | - ANC stands for Antenatal Care ,has been spelled out in page 22 |
|  | Sexual violence:   - Please provide further support for removing the category of rape. Provide additional support for this sentence: “The discussion led to the consensus that rape is not just an act of violence but a criminal offense, even though there have been reports of such actions by healthcare providers.” How did rape’s status as a crime fit into the discussion? What were the primary reasons laid out by the group for this item’s removal? - P. 23- Please clarify the sentence that begins with the phrase, “Some postnatal women…” Does this mean postnatal women suggested adding a new category: “to be forced to have sex with their partner during pregnant even when they are not feeling well”? Please explain, “claiming they are warm.” | - Thank you for your thoughtful review on the issue of rape. - After consulting various legal frameworks, engaging with legal professionals within our context, and conducting further comprehensive literature review, all authors have reached a consensus to retain rape as a form of obstetric violence within the category of sexual violence as there have been reported cases of such incidents within our context .This classification applies when rape is perpetrated against a pregnant woman without her consent, whether in community settings or healthcare facilities . The rape item has been added in table 4 page 20 and more explanation with reference have been added in the discussion on page 27-28 - This was not suggested by postnatal women but during the face validation with them, they agreed this to be common and added more information to strengthen it .They said sometimes Partners may pressure them by asserting that sexual pleasure is enhanced during pregnancy - This paragraph has also been rephrased for more clarity. Page 23-24 |
|  | Psychological/emotional violence:   - P. 23- Please add the word “differently” to the following item: “treating a woman (differently) based on her age, parity and marital status…” - P. 23- Please clarify this phrase, “experts and postnatal mothers added teenagers and high parity women as they are so vulnerable to violence during care provision at the health facilities in our settings.” Does this mean additional items based specifically on age and parity were added to the prototype? | - The word ‘differently’ have been added . Page 24 - Yes teenagers and high parity women were added to the prototype during the validation process, Based on experts experience it was agreed these two groups to be among the vulnerable groups for violence while pregnant and during labor. Page 24 |
| **7** | **Discussion** |  |
|  | - P. 26- Please clarify the sentence beginning with, “Experts also recommended the inclusion Health…” It is difficult to follow. | - “Health system conditions and constraints”, this category was derived from the World Health Organization (WHO) new evidence on mistreatment of women during childbirth. This document, was also one of the references used during the workshop. So during the plenary discussion experts suggested to be added as some of the listed items in this category, were also common in our settings. The whole paragraph has been rephrased too .Page 28 |
| **8** | **Strength and limitation** |  |
|  | - P. 27- Add a strength that highlights the practical utility of this OV prototype—what can it do, how can it advance the field? | - The strengths that highlights the practical utility of Ov in our settings ,have been added in page 28 |
|  | - P. 27- Please clarify this limitation. Is there any reason to assume that male partners and/or religious leaders will have more to say on OV than your current participants? | - Thank you for highlighting this important aspect of our research. The current participants offer invaluable first hand insights that significantly contribute to the depth of our findings. We also felt it was crucial to involve male partners and religious leaders in the validation process to ensure that their perspectives gathered during the qualitative phase, were properly represented. Their involvement could possibly allow them to assess the clarity of the language used, ensure that words were appropriately chosen , and incorporate any additional information that might not have been fully expressed during the interviews in the community. - However, this was not a major concern, as we already had a strong representation of women and experts, who are also integral members of the community. So this limitation has been cancelled from the main manuscript. |
|  | - Consider including a limitation that addresses the difficulty of including all perspectives in one prototype for OV. - How did you resolve scenarios of disagreement among your participants? - How did you ensure that all of the voices in the focus groups were heard? Is it possible that relations of power played out in the focus groups, such that some voices were louder and therefore had outsized impacts on the resulting model? | - This limitation have been added in page 29 - When disagreements arose, researchers encouraged open dialogue by asking clarifying questions and prompting participants to elaborate on their viewpoints. - Additionally, we established ground rules at the beginning of the workshop that emphasized respect for differing opinions. - To ensure that all voices were heard in our focus groups, we utilized a cyclic format/rotating speaking during discussions, allowing each participant an equal opportunity to speak without interruption and to directly prompt quieter members to share their views, ensuring that no one was overlooked during the discussions. - Furthermore, we provided anonymous feedback mechanisms such as written notes, so that quieter participants could share their thoughts without feeling pressured by dominant voices in the room. |
| 9 | Conclusion |  |
|  | - P. 27- Please lay out some of the more specific practical implications of your study—how can this be used to help pregnant people? To help healthcare providers? To address maternal mortality? - Please lay out the next steps for researching this topic—what are the important questions that remain about OV in this context? What are the important areas for future research? | - More specific practical implications for this study such as t implications to pregnant women, health care providers and how it can be used to address maternal mortality have been added ni page 29-30 - Recommendations for future research and important areas to concentrate on have been added in page 30 |

**Reviewer #2: Thank you for reviewing our manuscript we appreciate your comments**

| **SN** | **Comments** | **Response** |
| --- | --- | --- |
| **1** | **Abstract** |  |
|  | Results: Seven categories of obstetric violence components were identified, including physical violence, lack of supportive care and treatment, subjugation care, Unfavorable care environment, sexual violence, verbal violence, emotional and psychological violence. In addition, 24 subcategories of obstetric violence were also identified  **Issue:** I suggest the removal of the space between identified and the full-stop | - This has been corrected |
| **2** | **Funding details** |  |
|  | The word doctoral is misspelled | - This has been corrected in page 32 |
| 3 | **Introduction:** |  |
|  | Without acknowledging local expressions and interpretations of obstetric violence, there is a risk of underreporting or over-reporting the incidences prohibiting effective efforts to address it .  **Issue:** Kindly check whether the word “prohibiting” is correctly used in this context. | - The whole paragraph has been edited .Page 4 |
| **4** | **Study Context** |  |
|  | Kindly check citation 13. I think it is misplaced  Dodoma a capital City of Tanzania with a population of 3,085,625 million people (14). **Issues:** The sentence needs restructuring for clarity | - This has been corrected in page in page 4 |
| **5** | **Results** |  |
|  | Table 4: Final contextual components of obstetric violence after validation -Being chased from home or forced to be married forced  **Issue:** The second word, “forced” to be deleted | - The repeated word has been deleted .Page 21 |
| **6** | **Discussion** |  |
|  | This view was also reported in other study . **Issue:** The word ‘study’ to be replaced with the word ‘studies’ | - The word ‘study’ has been corrected to’ studies’. Page 25 |
| **7** | **Strength and imitation** |  |
|  | **Issue:** An l to be added to the word imitation to read limitations. The who statement to replaced with the title, ‘Strengths and limitations | - The word limitation has been corrected .Page 28 |
| 8 | **Conclusion** |  |
|  | I agree that the article is presented in an intelligible fashion and is written in standard English. However, I suggest that the authors consider correcting minor errors that I have highlighted above | We appreciate this recognition and your constructive comments .The whole document has been edited for grammatical issues |
