# Supplementary material for: Development and validation of context-specific components of obstetric violence: Experiences from the central zone of Tanzania
Source: PLoS One. 2025 Jun 18;20(6):e0326362. doi: 10.1371/journal.pone.0326362 (PMC12176237; doi:10.1371/journal.pone.0326362)
Supplement: Attachment — Submitted filename: RESPONSE TO REVIWERS COMMENTS.docx [file pone.0326362.s005.docx]

POINT BY POINT RESPONSE TO EDITOR AND REVIWERS COMMENTS

Dear editor, we would like to appreciate the comments given by you and reviewers on the manuscript titled “**Development and validation of context-specific components of obstetric violence: Experiences from the Central Zone of Tanzania*”*** as indicated in the tables below together with the amendment made:

**Reviewer #1: Thank you for reviewing our manuscript and for the constructive comments**

| **SN** | **COMMENT** | **RESPONSE** |
| --- | --- | --- |
|  | **Introduction** | |
| 1 | • This work could still benefit from a careful revision to remove unnecessary (e.g., the dash in first sentence: “widespread issue-affecting”) and add needed (e,g., you are missing a space and a period here: “reported to be between44% and 52%, In Tanzania”) punctuation. | -Dear reviewer, thank you for your constructive comments .  -The whole document have been revised more for grammatical errors and removing un necessary spaces and dash, we have also consulted the English language editor for proofreading and editing |
|  | • P. 4- first sentence: please explain what you mean by “normalizing it.” | -We appreciate your thoughtful comment.  -When we say, "normalizing obstetric violence, it means certain disrespectful childbirth practices become perceived as routine or even anticipated by some women that they will come across those acts as usual. This may occur due to repeated exposure, a lack of information about one's rights and a lack of adequate alternatives for access. As a result, some women normalize these practices as routine .We have also added more clarification in the manuscript in page 4 |
|  | • P. 4- please add the word “national” to: “Tanzania’s national guidelines have outlined | -The word “national” has been added .Page 4 |
| 2 | **Materials and methods** | |
|  | • P. 4- Please add a period and separate these into two sentences: “…experiences of obstetric violence at the facility level. As a result, some women avoid facility birth and then… | -The sentence has been revised for clarity. Page 5 |
|  | Phase I: The Qualitative Study  • Please explain who “ten-cell leaders” are. | -Ten-cell leaders are grassroots administrative leaders in Tanzania who build close relationships with household. Ten-cell leaders' understanding of family relationships, domestic issues and the local government, positions them to be knowledgeable and speak about their community.  -We have also added clarification in the manuscript in  page 7 |
| 3 | **Results** | |
|  | Verbal violence:  • P. 24- For this phrase: “especially if a woman fails to push the baby in a timely manner,” please add some context, such as “from the perspective of the healthcare provider.” You could indicate that healthcare providers have schedules/timelines in mind that have no correlation to the pregnant person’s health or wellbeing.  • P. 24- Also, please fix typo: “most common reason for why women hesitate to seek care” | -Thank you for this insight  -We have added more clarification on this paragraph as suggested and the typo have been fixed. Page 25 |
| **4** | **Discussion** | |
|  | • P. 26- fix typo: “components were valid and culturally acceptable”  • P. 26-27- Please consider adding one more sentence to the paragraph on the use of a syringe to manipulate nipples. What is the value of this exploration? Perhaps something that highlights the importance of collecting the full range of perspectives on these procedures in addition to collecting the medical communities’ perspective | -We appreciate this useful suggestion. We agree that highlighting the need to capture a diversity of perspectives enhances the discussion.  - An additional sentence has been included to bring out the need to learn about how such procedures are perceived not only by healthcare providers but also by the women who experience them and which is significant in informing respectful and culturally sensitive maternal care practices.Page 28 |
|  |  |  |

**Reviewer #2: Thank you for reviewing our manuscript we appreciate your feedback**

| SN | Comments | Response |
| --- | --- | --- |
| 1 | -Congratulations on the work you have done so far, and I am glad to review it .  -My comments in the last round of review have been addressed. However, after going through the document, I noted that there are issues of grammar and punctuation that need to be addressed. If the issues that I have raised are addressed, then I believe this great work will be ready for publication. Issues that I have noted in my reading are indicated hereunder | Dear reviewer ,we appreciate this recognition and insightful feedback  We have gone through the whole document and work on the grammatical issues and punctuation with the help of English language editor for proofreading and editing |
| 1 | **Introduction** | |
|  | Globally, estimates of obstetric violence range from 15% to 99% (3) with prevalence in Sub-Saharan Africa reported to be between44% and 52%, In Tanzania, obstetric violence remains a significant concern (4,5). – Page 3.  Issue  Space needed between the word ‘between and 44% | -The space has been added . Page 3 |
|  | Despite ongoing research in examining the definition, root causes and methodologies for assessing obstetric violence occurring during pregnancy, childbirth and after childbirth, there is no universal consensus on how to define and measure obstetric violence (8).Cultural and… Page 3.  Issue  Space between the full stop at citation 8 and Cultural needed. | -The space has been added. Page 4 |
|  | Tanzania’s guidelines have outlined the typology of disrespect and abuse in maternity care such as non-dignified care, abandonment, physical abuse, non-confidential care and non- consented care (10), yet they large draw from external context that may not fully reflect local realities (8,9,11). Page 4  Issue  The word “large” should be replaced with the word “largely” | -The word “large” has been edited . Page 4 |
|  | Comprehensive Emergency Obstetric and New-born Care (CEmONC) (13,14) Page 4.  Issue  Full stop needed at the end of citation 13 and 14. | -A full stop has been added . Page 4 |
|  | Maternal health care provision remains a major challenge in developing countries such as Tanzania resulting in ongoing high maternal mortality (15) .One major risk factor… page 4  Issue  Full stop needed at the end of citation 15 needed and spacing before the word, “One” | -A full stop and space have been added. Page 4 |
|  | Factors linked to home births in Tanzania encompass the hesitancy to seek care at a birthing facility due to individual and community experiences of obstetric violence at the facility level as a result , some women avoid facility birth and then experience preventable birth complications hence contributing to prevailing high maternal and neonatal mortality (17) .  Issue  Space between the word result and some to be deleted.  space between citation 17 and full stop to be removed. – check the rest of the citations for similar citation issues for the rest of the text.- | -The space has been removed . Page 5 |
|  | This study was conducted in two regions in the central zone of Tanzania from July 2023 to June 2024 .  Issue  space between citation between 2024 and full stop to be removed. | -The space has been removed |
| **2** | **DISCUSSION** | |
|  | Obstetric violence is globally recognized as a serious issue threatening maternal health and  well-being, but there is no consensus on how best should be defined due to cultural and  geographical differences (18).  Issue  The word “it” to be inserted between best and should… | -The sentence has been rectified . Page 26 |
|  | Furthermore, feedback from maternal health experts, health care providers and postnatal mothers during the validation process were utilized and incorporated whereby some items were modified .  Issue  The space between modified and full stop to be removed | -The sentence has been revised and space removed . Page 26 |
|  | These results align with a study conducted in the West Bank Palestine, where obstetric violence measurement tools were developed and validated, achieving a content validity index greater than 0.83 (41) indicating that all the components were valid and cultural acceptable .  Issue  The space between the word acceptable and full stop to be removed | -The space has been removed as seen in page 27 |
|  | Significant spiritual element of the childbirth experience (45).  Issue  Space between citation 45 and the full stop to be removed | -The space has been removed |
|  | There was particular concern about managing mentally distressed women during labor, considering staffing constraints.  Issue  Letter ‘a’ to be inserted between the words “was” and “particular” | -The letter ‘a’ has been inserted . Page 28 |
|  | … involves non-consensual sexual acts against pregnant women whether in community or healthcare settings (51–53)  Issue  Full stop needed after citation 51-53. | -A full stop has been inserted. Page 29 |
|  |  |  |
